# Social Capital and Preferences for Aging in Place Among Older Adults Living in Rural Northeast China

**DOI:** 10.3390/ijerph17145085

**Published:** 2020-07-14

**Authors:** Nan Lu, Shicun Xu, Qinghong Zhou

**Affiliations:** 1 Department of Social Work and Social Policy, School of Sociology and Population Studies, Renmin University of China, Beijing 100872, China; nalv9728@ruc.edu.cn (N.L.); 2019103845@ruc.edu.cn (Q.Z.); 2 Sau Po Centre on Ageing, The University of Hong Kong, Hong Kong, China; 3 Department of Population, Resources and Environment, Northeast Asian Studies College, Jilin University, Changchun 130012, China

**Keywords:** social capital, aging in place, older adults, rural China

## Abstract

The present study examines the association between cognitive social capital and structural social capital and aging-in-place among older adults living in rural Northeastern Chinese communities. Data were derived from a survey conducted in Dongliao County, Jilin Province, China, in late 2019. A quota sampling approach was used to recruit 458 respondents aged 60 years and older. Structural equation modeling was applied to test the proposed model. The results show that the measurement models of cognitive social capital and structural social capital were established in rural Chinese communities. Structural social capital was found to have a higher effect on aging in place than cognitive social capital (structural social capital: β = 0.241, *p* < 0.001; cognitive social capital: β = 0.118, *p* < 0.05). The findings highlight the crucial role both cognitive and structural social capital play in affecting the preference for aging in place among older rural Chinese adults. Policy and intervention implications are discussed.

## 1. Introduction

Aging in place has been defined as older adults’ capacity to remain in their own homes and local communities as they age, despite age- and health-related changes that might lead to higher levels of long-term care needs and dependence in regard to performing activities of daily living [1]. The majority of older adults prefer to live in their own homes, which not only provides them with a sense of belonging and security in relation to their homes and local communities but also allows them to fulfill their social roles and obligations [1]. Given the relatively high cost of institutional care, aging in place is recognized as an effective strategy to not only promote the quality of life of older adults but also to reduce the financial cost to individuals, families, and governments. Therefore, it is important to examine the social determinants of the preference for aging in place among older populations. 

Individuals’ socio-demographic characteristics, physical health decline, changes in family composition (e.g., widowhood and divorce), and financial status were found to be important determinants of aging in place [2,3]. Furthermore, aging in place is influenced greatly by the physical environment of the community (e.g., aged-care services and amenities) and individuals’ social ties to local communities [1,2,3]. On the one hand, older adults might need supplementary daily care and medical services to help them to continue to stay in their homes as they age. On the other hand, older adults tend to continue to engage in social involvements in their local communities, which provide a sense of autonomy, independence, social identity, and meaning in life. The literature on aging in place mainly focuses on physical environment and social services in the community. Meanwhile, there is a growing interest in community-based social capital and its influence on aging in place [1,3,4,5,6]. However, there are two major research gaps as follows. First, the conceptualization and measurement of social capital are not consistent in the literature. Many studies have used a single indicator to assess the multi-dimensional concept of social capital. In this study, we considered social capital as a latent construct, which cannot be observed directly but can be examined through a set of observed variables [7]. As compared to a single indicator, the latent construct approach is a more comprehensive and reliable instrument to assess social capital [7]. Second, given the nature of the two-tier society under study, the association between social capital and aging in place might vary across rural and urban communities in China. On the one hand, social capital tends to be more affluent in high-income communities, which can be reflected by higher levels of trust, social participation, and citizenship activities [8]. On the other hand, the presence of social capital might play a more salient role in enhancing the welfare of older residents living in economically and socially vulnerable communities, especially for those with relatively limited financial resources and low educational attainments. A recent systematic review suggested that a strong sense of familiarity and belonging in the community and social participation could outweigh the negative aspects of rural residence (e.g., limited access to transportation) and play important roles in achieving successful aging and aging in place in rural communities [9]. Therefore, it is important to conduct aging in place studies in economically underdeveloped regions and collect local evidence for the sake of policy and intervention development. While social capital has been tested in urban Chinese communities [10,11,12,13], there is a lack of relevant research in rural Chinese communities. Therefore, the present study aimed to establish a latent construct of social capital in a rural Chinese community context and examine the association between social capital and aging in place among older adults living in rural China. 

### 1.1. Social Capital and Aging in Place

Robert D. Putnam’s conceptualization of social capital is recognized as the most adopted definition in the health research field. From a collectivist perspective, Putnam conceptualized social capital as “features of social organization, such as trust, norms, and networks, that can improve the efficiency of society by facilitating coordinated actions” [14]. Social capital can also be defined from an individual perspective; in this context, social capital can be conceptualized as a form of capital that exists in both informal exchanges with others and social participation in formal organizations [15]. Communities are important places for the above social exchanges to take place among older populations. Coleman conceptualized social capital as social resources embedded in one’s social connections, where people share common memberships, reciprocity, trust, and social norms and values [16]. Social capital can be used to facilitate cooperation and pursue individual and collective benefits [15,17]. Lin [18] made a significant contribution to social capital theory by examining the role of bridges in information transfer and exchange across different social networks. 

Furthermore, social capital can be measured from cognitive and structural dimensions. Specifically, the cognitive dimension refers to an individual’s subjective perceptions in terms of trust and reciprocity in the community [19]. The structural dimension refers to relatively objective measures in terms of an individual’s social involvement in the community, such as organization membership, volunteering, social participation, and citizenship activities [19,20]. Social capital embedded in these weak ties enhances older adults’ capacity to access care and services, social interactions, informal and formal helping, and collaboration, which might help older adults to remain in their homes as they age [21]. For example, trust in the local community might enhance feelings of security and promote reciprocity in the neighborhood. These could be useful resources for older adults to meet their daily care needs. Social engagement in neighborhood and community activities might also provide a sense of belonging and comfort. 

Relatively high levels of social capital imply that local residents are willing to provide support to those who are older, frailer, and need assistance with activities of daily living when necessary. The social capital embedded in weak ties in the community is particularly useful in rural communities with limited financial resources and social infrastructures. Therefore, low levels of trust and reciprocity among neighbors and social involvements in formal organizations in local communities might leave older adults living in low-income rural communities at higher risk of social isolation, morbidity, and mortality. High levels of social capital can generate important social resources and provide environmental contexts in which older residents can remain in their homes, despite adverse conditions such as low income and poor social infrastructure.

Community-based social capital (hereafter, social capital) is considered as an important social determinant of aging in place [3,21]. However, the findings are mixed in the literature. A sense of belonging, perceived safety, and familiarity with local communities, for example, was found to be significantly associated with aging in place [1,22]. Information regarding home- and community-based services and personal care, as well as the levels of social engagement in the community, were found to be significant factors regarding aging in place among community-dwelling adults in the United States [4]. A recent Chinese study found that social capital indicators (e.g., trust in the local community and organization membership) were significantly associated with aging in place among older adults living in urban China [3]. However, by using a name generation method, Lum et al. [5] found that instrumental social support from neighbors, friends, and formal organizations was not significantly associated with aging in place among older Chinese adults living in low-income public housing estates in Hong Kong. These mixed findings might result from the lack of consensus in terms of the conceptualization and measurement tools of social capital, and the differences in the social and cultural contexts from which the samples were drawn. How community-based cognitive social capital and structural social capital influence the preferences regarding aging in place among older adults living in rural Chinese communities remains unknown. 

### 1.2. Population Aging and Filial Piety in Rural China

The number of older adults aged 65 years and older had exceeded 166 million in China by 2018 [23]. The urbanization rate in China also increased to more than 59.58% in 2018, indicating that a large proportion of older adults will continue to live in rural regions in the next few decades [23]. While family-based support systems are still the main source of support for older Chinese adults, the average family size has declined from 4.33 in 1950 to 3.00 in 2018 [23]. Filial piety is deeply rooted in Chinese culture and emphasizes adult children’s obligation to care for their older parents when necessary [24]. However, the declining average family size, the transition of living arrangements among older adults (changing from multi-generational households to skipped-generation and empty-nest households), urbanization and modernization have weakened rural Chinese families’ functions in supporting their older members. Furthermore, it is important to note that the increasing prevalence of rural-to-urban migration in China has led to millions of working-age adults moving to urban areas for more employment opportunities and better financial benefits, which has further widened the geographic distances across generations in rural regions. There are also increasing proportions of older adults living alone in rural China, as a consequence of divorce and widowhood [25]. Both widened intergenerational geographic proximity and living alone could decrease social connections among older adults. Finally, as discussed above, China is a two-tier society. Individuals with agricultural household registration status and those with non-agricultural household registration status, for example, have different levels of access to education and employment opportunities and health service benefits [26,27]. Furthermore, the health care and pension systems in rural China have undergone great reforms since early 2000s. The New Rural Cooperative Medical System, for example, has been implemented in 2003, and the coverage has increased dramatically to 86% in 2007 [28,29]. The New Rural Social Pension Scheme has been implemented in 2009 [27]. However, there is a large disparity in the benefits of rural and urban pension and health care systems [26,27,28]. Health resources (e.g., hospitals and health professionals) are also mainly located in urban communities [26]. The income gap between rural and urban residents has widened, rather than decreased, in the economic boom since 1978 [23]. Under such circumstances, social capital in the community plays a more important role in helping older rural adults remain in their homes and maintain social connections, especially for those with a low socioeconomic status. 

Based on the above literature review and theoretical framework, we hypothesized that both cognitive social capital and structural social capital would be significantly associated with the preference for aging in place among older adults living in rural Chinese communities, when sociodemographic characteristics, socioeconomic status, health conditions were controlled for.

## 2. Materials and Methods 

### 2.1. Sampling

Data were derived from a community survey that was jointly conducted by Renmin University of China and Jilin University in late 2019. A quota sampling method was used to recruit older respondents aged 60 years and older from Dongliao county, Liaoyuan city, Jilin Province, China. Ethical approval was obtained from the Ethics Committee of the University of Hong Kong (reference no. EA2003026). Liaoyuan city is located in the mid-southern part of Jilin Province. By the end of 2018, the local population numbered 1.17 million, around 21.92% of whom were aged 60 years and older (national average level: 17%). Around half of the local population have agricultural household registration status. Dongliao county surrounds the whole city of Liaoyuan, including 13 townships and 235 administrative villages. This region is thus suitable for studying social capital and aging in place among older populations in economically underdeveloped rural communities in China. 

The sampling procedures were as follows. First, 16 villages were randomly selected among the 235 villages in Dongliao county. Second, in each selected village, the survey team recruited 30 respondents aged 60 years and older according to referrals from the village commissions. The age and gender ratio of older respondents were consistent with the figures, based on the representative sample of Dongliao county in the latest national census. In order to be recruited for the survey, the respondents needed to have local household registration status, be aged 60 year and older, and have lived in local communities for more than 180 days in the past 12 months. 

A total of six trained interviewers conducted face-to-face interviews with older respondents at local community centers and their homes. Informed consent forms were obtained before the data collection. Age and gender ratio of the respondents were consistent with those from the local representative sample from the latest national consensus. The questionnaire collected rich information about the respondents’ sociodemographic characteristics, physical health, mental well-being, socio-economic status, family conditions, aging in place, and social capital. No personally identifiable information was collected. A total of 482 out of the 486 respondents successfully completed the survey interviews. The response rate is 99%. The Short Portable Mental Status Questionnaire (SPMSQ) was used to assess the respondents’ cognitive function [30]. In this study, we excluded those who did not pass the cognitive test, generating a final analytic sample of 458.

### 2.2. Measurements

#### 2.2.1. Outcome Variable

The outcome variable was the preference for aging in place. The respondents were asked whether or not they would prefer to continue to live in the local rural community. The answers were assessed by a binary variable (0 = no; 1 = yes). This approach has been widely used in the literature [3,5]. 

#### 2.2.2. Social Capital Variable

Eight indicators from the Short Social Capital Assessment tool and the World Bank’s social capital questionnaire were selected to assess social capital [20,31]. Specifically, four trust and reciprocity variables were used to represent the latent construct of cognitive social capital. The respondents were asked whether or not they agreed with the following four statements: “The majority of local residents living in this rural community can be trusted” (i.e., trust in the community); “the residents in the village community help one another out” (i.e., perceived helpfulness of others); “the local community is a big family and you consider yourself to be a member of this family” (i.e., feelings of belonging); “local residents care about not only their own benefits, but also others’ interests” (i.e., willingness to cooperate with others). The responses were assessed on a five-point Likert scale (1 = strongly disagree; 3 = neutral; 5 = strongly agree). 

Furthermore, four indicators were used to represent the latent construct of structural social capital, including the number of organization memberships, social participation, volunteering, and citizenship activities. Specifically, the respondents were asked about whether or not they had held memberships with various organizations in the past 12 months, including political parties, religious groups, women’s groups, sports groups, professional associations, charity groups, neighborhood committees, and community associations. The responses were assessed using a binary variable (0 = no; 1 = yes) and further summed to represent the number of organization memberships held (range: 0–9). Furthermore, the respondents were asked how frequently they had participated in social activities organized by those organizations in the past 12 months (ranging from 1 = never to 6 = more than twice per week), whether or not they had participated in the volunteering activities organized by those organizations in the past 30 days (0 = no; 1 = yes), and whether or not they had collaborated with other residents to solve a common issue in the past 12 months (0 = no; 1 = yes). 

#### 2.2.3. Covariates

The covariates included age, gender, marital status, number of children, living alone, educational attainments, income, activities of daily living, and self-rated health. Age was assessed in years. Gender, marital status, living alone, and educational attainments were assessed using binary variables (0 = men, 1 = women; 0 = other marital status, 1 = married; 0 = living with others, 1 = living alone; 0 = illiterate, 1 = primary school education or higher). The respondents were asked to report their annual household income and how many living children they had. Self-rated health was measured by a single question: “How do you evaluate your health condition?” The responses were further recoded as a binary variable (0 = very poor/poor/fair; 1 = good/very good). Finally, the 10-item Barthel Index was used to assess activities of daily living among the older respondents [32]. The responses were assessed on a 10-point scale, ranging from 0 (very difficult and unable to complete the task independently) to 10 (no difficulty). Summed scores were calculated to represent the dependence levels regarding activities of daily living (ADL; range = 0–100). 

### 2.3. Data Analysis

In the present study, we used structural equation modeling to test the proposed hypotheses, using Mplus 7.0 [7,33]. A two-step approach was applied. First, the measurement model of cognitive social capital and structural social capital was established using confirmatory factor analysis. The following fit indexes were used to assess the model fit: a chi-square test, the weighted root mean square residual (WRMR), the comparative fit index (CFI), the Tucker–Lewis index (TLI), and the root mean square error of approximation (RMSEA). Non-significant chi-square values and CFI and TLI estimates higher than 0.95, RMSEA values lower than 0.05, and WRMR values lower than 1.00 indicate adequate model fit [7,34]. Second, a structural model was established to test the relationship between social capital and aging in place, with socio-demographic characteristics, socio-economic status, and family and health conditions controlled for. Research data can be found in the Supplementary Materials, File S1.

## 3. Results

### 3.1. Descriptive Statistics

Sample characteristics are illustrated in Table 1. The average age of the respondents was 69.41 years. Around half of the respondents were older men, 70.3% were married and lived with their spouses, and 15.7% lived alone. Moreover, 36.9% of the respondents were illiterate, and nearly half reported that their annual household incomes were lower than RMB 10,000. A total of 41.7% reported that their health condition was very poor, poor, or fair. Of all the respondents, 85.8% reported that they had no limitations in their activities of daily living. A total of 74.9% preferred to continue to live in their local rural communities.

### 3.2. Structural Equation Modeling

In the first stage, we established a measurement model of cognitive social capital and structural social capital. The fit indexes indicated adequate model fit (χ^2^ (18) = 19.098, *p* = 0.3858, RMSEA = 0.012, CFI = 0.999, TLI = 0.998, WRMR = 0.495). The standardized factor loading estimates ranged from 0.671 to 0.927 for cognitive social capital, and from 0.390 to 0.915 for structural social capital. The details of the measurement model are illustrated in Table 2.

**Figure 1:**
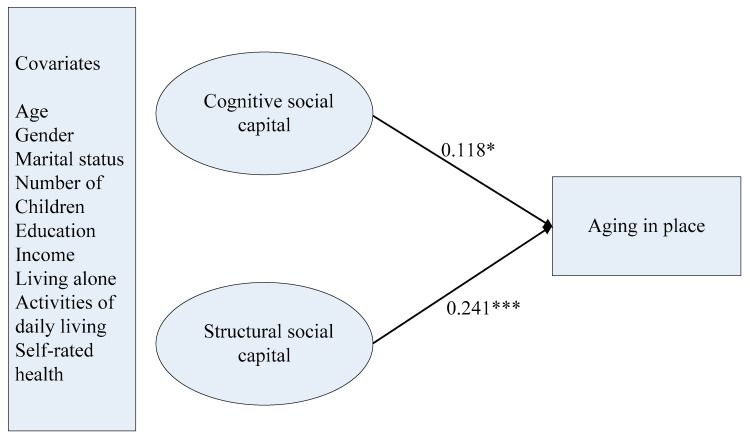
Final model of social capital and aging in place. *Notes:* Standardized coefficients are reported. * *p* < 0.05 (two-tailed); *** *p* < 0.001 (two-tailed).

In the second stage, we entered the aging in place variable and covariates in the final structural model. The fit indexes also suggested an adequate model fit (χ^2^ (78) = 84.961, *p* = 0.2760, RMSEA = 0.014, CFI = 0.992, TLI = 0.988, WRMR = 0.572). Cognitive social capital was found to be significantly associated with aging in place (b = 0.321, *SD* = 0.143, *p* < 0.05). Structural social capital was found to have a higher effect on aging in place (b = 0.496, *SD* = 0.138, *p* < 0.001). Furthermore, older respondents who were married and had some educational attainment and higher levels of self-rated health were more likely to prefer to continue to live in their local communities (marital status: b = 0.366, *SD* = 0.0180, *p* < 0.05; education level: b = 0.386, *SD* = 0.0139, *p* < 0.01; self-rated health: b = 0.518, *SD* = 0.0143, *p* < 0.001). Details of the final structural model are illustrated in Figure 1. For the relationships between covariates and social capital and aging in place are presented in Supplementary Table S1. 

## 4. Discussion

The present study is one of the first attempts to conduct latent constructs of social capital in rural Chinese contexts and further examine social capital and aging in place among older rural residents. The findings provide new evidence in terms of the application of social capital theory in rural Chinese contexts. The findings are also important for developing preventive strategies and social interventions for promoting aging in place and healthy aging in rural China. 

Consistent with the findings of previous studies [10,11,12], the findings of this study suggest that higher levels of cognitive social capital indicate higher levels of trust, reciprocity, and a sense of belonging to local communities, while higher levels of structural social capital foster higher frequencies of social participation, more organization memberships, and more volunteering and citizenship activities in rural China. Previous studies have indicated that some social capital indicators (e.g., a sense of belonging and familiarity, trust, and organization memberships) are significant factors of aging in place [1,2,3,4]. The findings of the study propose that the latent construct of structural social capital has a higher impact on aging in place than cognitive social capital does. This may be because both cognitive social capital and structural social capital provide important social supportive resources for older adults to facilitate their living conditions and represent important social systems through which older adults share memberships and values with other residents. Older rural Chinese adults often encounter a range of life challenges, such as the transition of filial piety, traditional family living arrangements, limited access to health resources, and poverty. Under such circumstances, formal supportive resources from organizations might play a more important role in meeting long-term care needs than informal supportive resources. Structural social capital may also play a salient role in low-income rural communities by providing access to important information, services, and care through formal organizations.

The policy and intervention implications of the findings of this study are as follows. First, social capital constructs are potentially effective instruments and are recommended to be included in the screening tools used to identify at-risk populations in rural communities. Future intervention strategies for older rural adults could also place greater emphasis on compensation for inadequate social networks. Social connections between older adults and social organizations can be used to promote access to services and social resources in the community in an efficient manner [6]. For example, local clinics and rural committees are important formal organizations through which older adults can receive support and have their social and medical needs met. Moreover, social capital interventions should focus on meeting the major social needs of rural older residents through enriching their social connections and resources, which could benefit older adults and their families and promote participation rates among rural residents. This strategy would not only promote intergenerational relationships but also help elderly individuals’ families to mitigate the burden of caregiving. Furthermore, interventions such as information sharing in regard to daily care services and long-term health, crisis interventions, peer support programs, and volunteering programs should be developed to achieve healthy aging [12], promote intergenerational solidarity [35], and encourage the young-old to care for old-old groups [6]. Finally, expanding access to health and service resources in the community is crucial for meeting the long-term care needs of older residents and enhancing the efficiency of social capital (e.g., transferring information and resources). Subsidies for home modifications among low-income older rural residents and tax incentives for community-based aged care services, for example, could be useful in regard to helping older residents to continue to stay in their homes safely and independently. Senior centers and service systems in rural communities should also be strengthened by developing and integrating aged-care service systems, relocating health and social resources to where older adults live, and facilitating older adults’ participation in formal organizations and citizenship activities.

This study has the following limitations. First, the data is cross-sectional in nature. Hence, the causal relationship between social capital and aging in place could not be examined in this study. Based on social capital theory, we have provided the theoretical rational for the proposed directions of the above relationships. Future longitudinal studies are needed to examine these casual relationships. Second, we did not use a random sampling method to recruit the respondents in this study. The empirical generation of the findings should therefore be made among populations with similar sociodemographic characteristics and social and economic backgrounds. Third, future studies should examine the interplay between physical environment and social capital in the community and their influences on aging in place. Finally, we did not examine the underlying mechanisms linking social capital to aging in place. For example, future studies should be conducted to examine the interplay among the sub-dimensions of social capital, as well as the potential mediator roles of social support and diffusion of information of health knowledge and service in the relationship between social capital and aging in place. 

## 5. Conclusions

The present study investigates the association between social capital and aging in place among rural older Chinese adults. The latent constructs of cognitive social capital and structural social capital were established in rural Chinese contexts. The findings show that both cognitive social capital and structural social capital play important roles in influencing older adults’ preferences in regard to aging in place, with the latter factor having a larger impact. The latent constructs of social capital can be used as screening tools to identify populations who are at-risk of having limited access to community supportive resources, which could lead to morbidity, mortality, and the need for institutional care. Social interventions should also be conducted to meet the social needs of older adults and their families and to further promote social capital in both informal and formal social connections in the community. 

## Figures and Tables

**Table 1:** Sample characteristics (*n* = 458).

Characteristics	*n* (%)	Mean (*SD*)
**Age**		69.41 (6.21)
60–69	270 (59.0)	
70 or above	188 (41.0)	
**Gender**		
Men	235 (51.3)	
Women	223 (48.7)	
**Married**	322 (70.3)	
**Education**		
Illiterate	169 (36.9)	
Primary school or above	289 (63.1)	
**Annual household income**		
Equal or less than RMB10000	241 (52.6)	
RMB10001 or above	217 (47.4)	
**Self-rated health**		
Very poor/poor/fair	191 (41.7)	
Good/very good	266 (58.1)	
**ADL**		98.08 (6.62)
**Number of Children**		2.42 (1.31)
**Living alone**	72 (15.7)	
**Prefer to continue to live in the community**	343 (74.9)	

Notes: ADLs = activities of daily living.

**Table 2:** Measurement model of social capital.

Factor Indicator	Estimate	*SD*	Standardized Estimate	*SD*
**Cognitive social capital**				
Trust in local community	1.000	0.000	0.671 ***	0.016
Willingness to cooperate with others	1.324 ***	0.057	0.927 ***	0.009
Perceived helpfulness of others	1.082 ***	0.066	0.840 ***	0.011
Feelings of belonging	1.265 ***	0.052	0.891 ***	0.009
**Structural social capital**				
Organization memberships	1.000	0.000	0.723 ***	0.723
Social participation	2.788 ***	0.498	0.915 ***	0.915
Volunteering	0.683 ***	0.141	0.390 ***	0.390
Citizenship activities	0.839 ***	0.119	0.479 ***	0.479

*Notes:* *** *p* < 0.001 (two-tailed).

## Supplementary Materials

The following are available online at https://www.mdpi.com/1660-4601/17/14/5085/s1. File S1: Research data; Supplementary Table S1: Final model of the relationship between covariates and social capital and aging in place.
