# Supplementary material for: Social Capital and Preferences for Aging in Place Among Older Adults Living in Rural Northeast China
Source: Int J Environ Res Public Health. 2020 Jul 14;17(14):5085. doi: 10.3390/ijerph17145085 (PMC7400209; doi:10.3390/ijerph17145085)
Supplement: Supplementary file 1 [file ijerph-17-05085-s001.zip › ijerph-854597-supplementary/ijerph-854597-supplementary Table S1.docx]

**Supplementary Table S1.** Final Model of the Relationship Between Covariates and Social Capital and Aging in Place.

|  | **Aging in Place** | | | **Cognitive Social Capital** | | | **Structural Social Capital** | | |
| --- | --- | --- | --- | --- | --- | --- | --- | --- | --- |
|  | **B** | **S.E.** | **β** | **B** | **S.E.** | **β** | **B** | **S.E.** | **β** |
| Age | −0.016 | 0.015 | −0.092 | −0.004 | 0.005 | −0.055 | −0.008 | 0.006 | −0.087 |
| Gender | 0.156 | 0.147 | 0.069 | −0.069 | 0.046 | −0.084 | −0.039 | 0.059 | −0.036 |
| Marital status | 0.366 | 0.180 | 0.151 * | −0.096 | 0.059 | −0.107 | 0.012 | 0.085 | 0.010 |
| Number of children | 0.057 | 0.067 | 0.066 | 0.015 | 0.020 | 0.048 | −0.024 | 0.033 | −0.059 |
| Education | 0.386 | 0.139 | 0.166 * | 0.002 | 0.046 | 0.002 | −0.027 | 0.060 | −0.024 |
| Income | 0.000 | 0.000 | 0.041 | 0.000 | 0.000 | −0.030 | 0.000 | 0.000 | 0.217 *** |
| Living alone | −0.233 | 0.211 | −0.077 | −0.126 | 0.066 | −0.113 | 0.150 | 0.106 | 0.102 |
| ADL | 0.008 | 0.013 | 0.043 | −0.0091 | 0.003 | −0.021 | 0.008 | 0.006 | 0.087 |
| SRH | 0.518 | 0.143 | 0.228 * | 0.085 | 0.045 | 0.102 | 0.037 | 0.061 | 0.034 |

* *p* < 0.05, *** *p* < 0.001.
